# Global Initiative for Chronic Obstructive Lung Disease (GOLD) 2023 Guidelines Reviewed

**DOI:** 10.2174/0118743064279064231227070344

**Published:** 2024-01-10

**Authors:** Munish Sharma, Sushil Joshi, Prakash Banjade, Shekhar A Ghamande, Salim Surani

**Affiliations:** 1 Division of Pulmonary, Critical Care and Sleep Medicine, Baylor Scott and White Medical Center, Temple, Texas; 2 Department of Medicine, Mantra Hospital and Research Center, Kanchanpur, Nepal. Nepalese Army Institute of Health Science, Kathmandu, Nepal; 3 Department of Medicine, Manipal College of Medical Sciences, Pokhara, Nepal; 4 Division of Pulmonary, Critical Care and Sleep Medicine, Baylor Scott and White Medical Center, Baylor College of Medicine, Temple, Texas; 5 Adjunct Clinical Professor of Medicine, Texas A and M University, Texas, United States

**Keywords:** Chronic obstructive lung disease, Bronchoscopy, Computed tomography chest, Global initiative for chronic obstructive lung disease, Inhalers, Preserved ratio impaired spirometry

## Abstract

The Global Initiative for Chronic Obstructive Lung Disease (GOLD) report is an essential resource for all clinicians who strive to provide optimal care to patients with chronic obstructive lung disease (COPD).

The annual report of GOLD makes few revisions and updates besides including data from the preceding year. At an interval, GOLD comes up with a significant modification in its guidelines, which is generally a major overhaul of the pre-existing guidelines. According to the latest 2023 updates, published in November 2022, there have been significant advancements made in the field of COPD. These include the development of more precise definitions for COPD and its exacerbations, the introduction of a new set of parameters to measure exacerbation severity, and updating the COPD assessment tool. Additionally, revisions have been made to the initial and follow-up treatment guidelines. The report also simplifies the treatment algorithm and sheds light on new findings that suggest the use of pharmacological triple therapy can reduce mortality rates. Furthermore, the report includes discussions on inhaler device selection and adherence to COPD medications. These improvements demonstrate a continued effort to enhance COPD treatment and management. Although there are some areas that could benefit from more detailed guidance and explanation, such as the proper utilization of blood eosinophil counts for treatment decisions, and the establishment of treatment protocols post-hospitalization, the latest modifications to the GOLD recommendations will undoubtedly aid healthcare providers in addressing any gaps in patient care. We aim to highlight key changes in the GOLD 2023 report and present a viewpoint about their potential implications in a real-world clinical scenario.

## 1 INTRODUCTION

The global burden of COPD is anticipated to rise in the following decades due to an aging population and continuing exposure to risk factors [1]. Every year, the Global Initiative for Chronic Obstructive Lung Disease (GOLD) recom- mendations undergo updates to ensure that they are aligned with the latest developments and innovations. The important objective of the GOLD is to raise awareness about the impact of COPD and assist in its prevention and management across the globe. The GOLD report, which was initially released in 2001, offers scientifically backed guidance on COPD diagnosis, stable disease management, handling of exacerbations, and comorbidities [2]. Healthcare professionals following GOLD recommendations may improve COPD outcomes and reduce resource utilization [3, 4]. We want to highlight major changes in the GOLD 2023 guideline, which is in its 6^th^ edition now [5].

## 2 THE NEW DEFINITION OF COPD: WHY WE NEEDED IT?

GOLD now defines COPD as “a heterogeneous lung condition characterized by chronic respiratory symptoms (dyspnea, cough, expectoration, exacerbations) due to abnormalities of the airways (bronchitis, bronchiolitis) and/or alveoli (emphysema) that cause persistent, often progressive, airflow obstruction” [5, 6]. The “heterogeneity” primarily urges us to expand our viewpoint beyond the notion of COPD being a single pathogenetic process related to smoking. Implications of developmental lung issues during perinatal and early childhood, recurrent infections, poorly controlled asthmatics, biomass fuel exposure, alpha-1 antitrypsin deficiency, and COPD of unknown cause stretch the “etiotypes” beyond the stereotypical focus on smoking only (Table **1**). These etiotypes may spur further research and result in therapeutic implications. GOLD has also embraced the concept of preserved ratio impaired spirometry (PRISm). The new definition is also comprehensive and includes a younger and increased proportion of female subjects, as shown in Table **1**.

### 2.1 FEV1, FVC and PRISm

To measure forced vital capacity (FVC) using spirometry, the patient must start by taking a deep breath. Afterward, they should exhale forcefully and for as long as they can manage. The amount of air exhaled during this maneuver is the FVC. The Forced Expiratory Volume in one second (FEV1) is the volume of air that can be forcefully exhaled in the first second of a Forced Vital Capacity (FVC) maneuver. This measurement is often lower in patients with obstructive respiratory diseases like asthma or emphysema. The FEV1/FVC ratio assesses whether the respiratory pattern is obstructive, restrictive, or normal [7].

The term PRISm has been proposed to identify patients who do not have Forced Expiratory Volume in the first second (FEV1)/ Forced Vital Capacity (FVC) < 0.7 post bronchodilation but have abnormal spirometry (FEV1 < 80% of reference post-bronchodilator) [8]. Almost a quarter of these patients develop fixed airflow obstruction in the succeeding 4-5 years, which could represent an opportunity to reduce disease [9, 10].

## 3 NEW SCREENING AND CASE-FINDING GUIDELINE

GOLD 2023 expands on screening recommendations. The presence of risk factors, such as more than 20 pack years of smoking, developmental issues with lungs, recurrent respiratory infections, or Symptoms indicative of COPD should undergo screening spirometry [11]. Questionnaires and peak flow meters have been proposed as potential adjunctive tools for screening purposes [12]. Data on the use of spirometry for screening and early diagnosis in children and young adults at risk of COPD due to poor lung development have been mixed.

### 3.1 Value of Computed Tomography (CT) Chest in COPD

The presence of emphysema in CT chest aids in diagnosis when spirometry is not diagnostic, and it can predict a rapid decline in FEV1 and indicate a higher risk of malignancy [13]. Detection of bronchiectasis in COPD, which is reported to be around 30% on CT chest, also predicts recurrent exacerbations and higher mortality [6, 14]. Lung cancer screening guidelines with low-dose chest CT scans increase emphysema detection and characterization. CT chest also helps in lung volume reduction surgery (LVRS) or endobronchial valve planning.

However, there is no role for routine CT chest scans in all patients with COPD.

### 3.2 Alterations in ABCD Assessment Tool: Major Implications

**Fig. (1):**
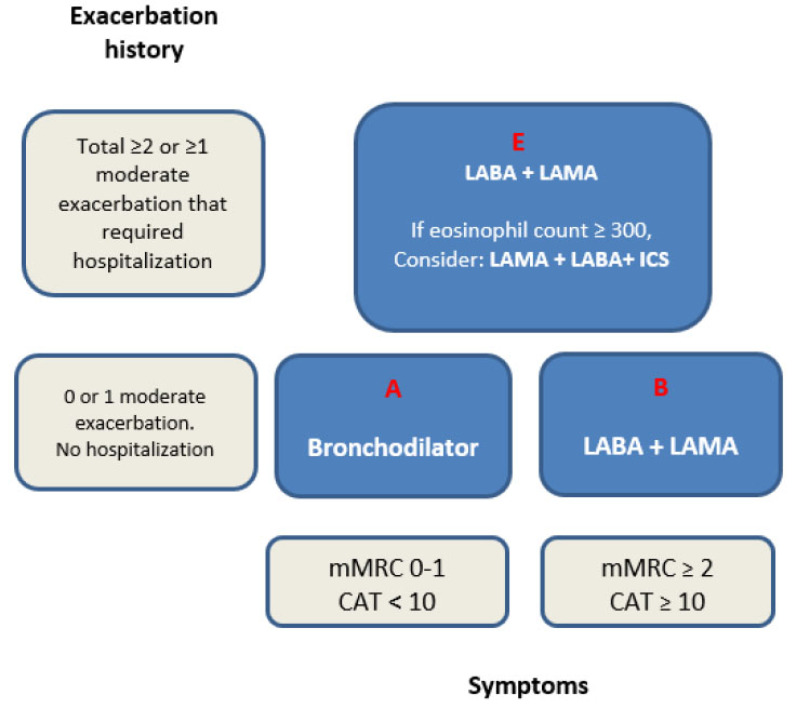
Shows a simple recreation of ABE assessment tool and pharmacological options based on 2023 GOLD guidelines.

ABCD tool, included in the GOLD guideline prior to 2023, combined FEV1 with measures of disease severity that included episodes of COPD exacerbations, hospitalization, and patient functional status [15]. It has laid more emphasis on the “treatable” traits of COPD, such as the burden of symptoms and exacerbation history (Fig. **1**).

GOLD 2023 guideline has now phased out categories C and D in the assessment tool and replaced them with a single category E. Patients with no exacerbation or only one exacerbation not leading to hospital admission fall into category A or B. Patients are categorized as A if their mMRC scale is 0-1 or their COPD assessment tool (CAT) score is less than 10. Patients fall into category B if their mMRC scale is 2 or more or their CAT score is 10 or more [16]. This has not changed as compared to previous guidelines. Patients in category A have mild symptoms and typically should be managed with bronchodilators. Patients in category B are more symptomatic and treatment recommendations include dual long-acting bronchodilator therapy (long-acting beta-2 agonist, LABA/ Long-acting muscarinic antagonist, LAMA). Category E includes all patients with a history of two or more exacerbations or one or more exacerbations leading to hospitalization. (Fig. **1**) [5]. The more severe Category E patients should be started on dual bronchodilator therapy (LABA/LAMA). If the blood eosinophil count is ≥ 300 cells/microliter, then the recommendation is to consider a triple therapy combination of LABA/LAMA and Inhaled corticosteroid (ICS) (Fig. **1**).

Two landmark studies influenced the management of GOLD group E patients.In the Informing the Pathway of COPD treatment (IMPACT) trial, triple inhalers (LABA/LAMA/ICS) decreased moderate to severe COPD exacerbations by 25 and 15% annually compared to LABA/LAMA and LABA/ICS, respectively [17]. The study also showed 54 ml (on average) improvement in FEV1 at the 12-month endpoint compared to LABA/LAMA and 97 ml compared to LABA/ICS. 42% of patients on triple inhalers had a four or more-point decrease in St. George's Respiratory Questionnaire (SGRQ) compared to 34% each in patients on LABA/LAMA and LABA/ICS inhalers [17].

Thus, there was an overall improvement in quality of life with the triple inhaler. The Efficacy and Safety of Triple Therapy in Obstructive Lung

The disease (ETHOS) trial also tested triple inhalers with LABA/LAMA and LABA/ICS. The annual moderate to severe COPD exacerbation rate was 1.07 in the low-dose triple inhaler group compared to 1.42 in LABA/LAMA and 1.24 in LABA/ICS group [18].

A notable change in the new guidelines is the elimination of groups C and D, including the more severe patients. There was a likelihood of misclassification of patients to group C based on improper perception and reporting of symptoms by the patients [19]. Thus, the abolishment of categories C and D and the creation of E have lumped patients with greater symptom burden and exacerbation risk under the same umbrella. This facilitated streamlining the correct use of long-acting bronchodilators and inhaled corticosteroids in COPD. ICS/LABA combination now does not play any role in patients with COPD without asthma. Real-life experiences using these recommendations will inform us about the impact of these significant changes, but they also raise important questions.

(a) What would be the recommendation by GOLD for resource-limited countries and communities where provision for even essential medicines is still a challenge?

(b) What challenges would be with patents for manufacturing triple inhaler therapies for developing nations [20]?

(c) How widely will the paradigm shift from using ICS/LABA to only using LAMA/LABA or triple therapy be accepted?

### 3.3 Updates on COPD Exacerbation, its Management, Discharge and Follow-up

With the recognition that an exacerbation represents a major change in the disease trajectory, the 2023 guideline has come up with a new definition for the exacerbation of COPD. It is now defined as “an event characterized by dyspnea and/or cough and sputum that worsen over < 14 days. It may be associated with tachypnea or tachycardia” [21]. The notion of systemic or local inflammation instigated by an airway infection, pollutants, or other triggers has been implicated in COPD exacerbation. The emphasis is on the etiology and triggers of COPD exacerbation similar to the section in the definition of COPD in a stable patient. Addressing confounding factors such as pneumonia, pulmonary embolism, congestive heart failure, acute coronary syndrome, pleural effusion, and pneumothorax set treatment priorities.

Intervention for COPD exacerbation based on 2022 recommendations of mild, moderate, and severe classification has also seen a proposed change in the current guidelines. Scoring of different variables threshold that includes visual analog dyspnea scale, respiratory rate, heart rate, oxygen saturation, C-reactive protein, partial pressure of Oxygen, and partial pressure of carbon dioxide has been used to stratify the severity (Table **2**) [21]. There were no major changes in the treatment strategy for COPD exacerbations. This guideline has outlined follow-up recommendations for the first 1-4 weeks and 12-16 weeks of hospital discharge in COPD patients with spirometry (Tables **3** and **4**). Overall, the follow-up instructions should help reduce re-hospitalization and provide a better transition of care.

### 3.4 Bronchoscopic Interventions in COPD. Exploring New Horizon

It is well known that hyperinflation is a significant feature of COPD. It is established that lung volume reduction surgery (LVRS) improves chest wall and diaphragm mechanics by removing hyperinflated lungs in selected patients with COPD. However, LVRS can be associated with significant morbidity and mortality [22]. Endoscopic lung volume reduction (ELVR), encompassing means like endobronchial valves (EBV), sealants, and vapor ablation (VA), has been suggested by this report as a viable alternative to LVRS mainly due to less morbidity and mortality [22]. EBV is a device that prevents air entry during inspiration but allows the one-way escape of air and secretions during expiration, thereby promoting atelectasis of the hyperinflated part of the lung. Lung sealants such as a mixture of fibrin, polymer, and thrombin can collapse emphysematous areas. Vapor ablation is the direct application of steam vapor by a bronchoscope to induce inflammation and subsequent atelectasis of a lung segment.

A key concept emphasized is the interlobar fissure integrity, which prevents collateral ventilation while describing ELVR choices. If the fissure integrity is > 90% as determined by high-resolution CT chest, EBV, VA, sealants, or LVRS are options for treatment. If collateral ventilation is present, EBV is excluded, while VA, sealants, and LVRS can still be valid options in selected patients [23, 24]. Discussion about nitrogen cryospray (liquid nitrogen application to ablate abnormal epithelium and cause mucosal regeneration) and rheoplasty (pulsed electricity applied by an endobronchial catheter) are recognized as potential treatment options for chronic bronchitis in this current guideline. Similarly, targeted lung denervation to blunt the effect of the parasympathetic nervous system and decrease mucus production has been mentioned for patients with frequent COPD exacerbations [6].

We feel that advanced bronchoscopy in COPD can be an important additional resource for COPD patients not improving on optimal medical treatment and pulmonary rehabilitation. A significant investment in a multidisciplinary team infrastructure with trained interventional pulmonologists is necessary.

### 3.5 Pulmonary Rehabilitation. Can Telerehabilitation be an Effective Alternative?

Pulmonary rehabilitation consists of exercise training, education for the specific disease process, and learned interventions as a self-management tool [25]. Barriers to utilizing pulmonary rehabilitation include access, availability, and funding. The GOLD guidelines discuss current evidence on Telerehabilitation programs in COPD. In a systematic review published in 2022, ten studies (n=245) were evaluated. There were positive effects of telerehabilitation on functional exercise capacity, anxiety, depression, quality of life, and the overall impact of COPD on a patient's personal life. Interestingly, there was no overall impact on dyspnea [26]. A Cochrane review concluded that telerehabilitation was safe and achieved similar outcomes compared to in-person rehabilitation in patients with chronic respiratory diseases.

However, the limitations include a small number of studies with a high degree of heterogeneity [27]. GOLD 2023 guideline has determined that there is still a need for definite evidence for telerehabilitation.

There is a need for telerehabilitation to be standardized in terms of mode of delivery, exercise prescription, post-COPD exacerbation interventions, and optimal duration for the maximal derivation of benefit. Besides, the technology should be user-friendly and easily accessible.

### 3.6 COPD in the Era of Coronavirus Disease 2019 (COVID-19)

Due to the outbreak of COVID-19, routine diagnosis and subsequent management of COPD became a challenge. With limited regular in-person office visits, difficulties in performing pulmonary function testing (PFT), and medication shortages, the plight of COPD patients was multifold. GOLD 2023 has discussed the interplay between COPD and COVID-19 [28, 29].

Per GOLD, it is unclear if COPD predisposes patients to a higher risk of contracting severe acute respiratory syndrome coronavirus 2 (SARS-CoV-2). Even though the results are mixed, the GOLD report includes evidence indicating a higher risk of severe COVID-19 in patients with COPD [30]. After adjusting for the confounding factors, the data included in the GOLD report suggested that there is an overall higher mortality risk in COVID-19 patients with COPD (Odds ratio 1.41, 95% confidence interval 1.37 – 1.65) and intensive care unit admission (Odds ratio 1.25, 95% confidence interval 1.08 – 1.51) [31]. Poor compliance with maintenance therapy in COPD, decreased baseline pulmonary reserve, and difficult access to healthcare resources have been signaled as the predictors of poor outcomes. The guidelines recommend testing even for mild symptoms, considering other co-pathogens during COPD exacerbations, and being alert for relapses in these patients.

GOLD 2023 has suggested limiting PFT to urgent or essential cases for diagnosis of COPD, especially during high COVID-19 prevalence. Reverse-transcriptase polymerase chain reaction (RT-PCR) has been recommended whenever possible before performing PFT during an outbreak [32]. However, these recommendations don’t apply to the current COVID incidence and prevalence. Current evidence suggests continuing corticosteroids and inhaled bronchodilators during COVID-19 infection. The United States Centers for Disease Control and Prevention Advisory Committee of Immunization Practices (ACIP) recommendations for pneumococcal vaccination (pneumococcal conjugate vaccine [PCV] 20 alone or PCV15 followed by pneumococcal polysaccharide 23 vaccine) and yearly influenza vaccine recommendations would not change in COPD patients during COVID- 19 pandemic per GOLD report.

### 3.7 Limitations of GOLD 2023 Guidelines

According to the GOLD 2023 report, triple therapy should only be considered a first line of treatment for patients whose blood eosinophil count is ≥300 cells/μL and who have experienced two moderate or one severe exacerbation. Recent research suggests that patients with blood eosinophil counts between 100 and 300 cells/L, especially those who have been hospitalized and are already taking medication, may benefit from adding an ICS to their current treatment plan [33]. More study is required into the use of lower thresholds for triple therapy commencement.

Patients who experience exacerbations are now treated using a follow-up protocol that does not define the severity or frequency of the exacerbations. Clarity regarding how exacerbation severity should affect treatment choices is required, given the correlation between exacerbation severity and the risk of subsequent exacerbations and/or mortality.

Receiving treatment recommendations post-hospitalization would prove to be quite beneficial. Various studies highlight that hospitalizations tend to increase the likelihood of poor outcomes, subsequent hospitalizations and mortality [34, 35]. Research has shown that triple therapy significantly reduces the likelihood of future hospitalizations following moderate and severe exacerbations. The guideline needs to clarify the recommendations that regulate the utilization of triple therapy for patients who have undergone hospitalization due to exacerbation [17, 18].

## CONCLUSION

The 2023 GOLD report was awaited with enthusiasm in anticipation of some paradigm-shifting changes. We believe this report contains some important changes that are relevant not only for pulmonologists but also for general medical practitioners. Early diagnosis and prompt initiation of treatment have been kept at the center of this revised version. Heterogeneity and etiotypes in COPD, the definition of subtypes, the new ABE assessment tool, renewal of continued interest in COPD education and rehabilitation, advanced bronchoscopy interventions, and implications of COVID-19 in COPD patients in the past few years are some of the salient features that make this report important. We appreciate the herculean efforts made by the GOLD committee to improve the recognition and management of COPD and look forward to the impact of the new GOLD guidelines.

## Figures and Tables

**Table 1:** A simple tabular adaptation of etiotypes of COPD based on GOLD 2023.

COPD due to genetic causes such as alpha-1 antitrypsin deficiency and other genetic variants.
COPD due to abnormalities in development of lung during perinatal and early childhood. Example: premature birth, low birthweight.
COPD due to smoke exposure (active and passive tobacco use, vape or cannabis use). COPD due to biomass.
COPD due to infections in childhood, tuberculosis, *etc*.
COPD associated with Asthma.
Unknown cause of COPD.

**Table 2:** A simple adaptation of severity of COPD exacerbation from GOLD 2023 guidelines.

**Severity of COPD** **Exacerbation**	**Variables Used to Classify**
Mild	VAS dyspnea < 5, HR < 95 beats/minute, RR < 24 /minute, resting SaO_2_ ≥ 92 on room air or baseline oxygen, CRP < 10 mg/liter
Moderate	VAS dyspnea ≥5, HR ≥95 beats/minute, RR > 24/minute, resting SaO_2_ < 92%, CRP > 10, PaO_2_ ≤ 60 and/or PaCO_2_ >45
Severe	PaCO_2_ > 45 and pH < 7.35. Rest criteria same as those in “moderate” class

**Note: **VAS: Visual analogue dyspnea scale, RR: Respiratory rate, HR: heart rate, SaO_2_: oxygen saturation, CRP: C-reactive protein, PaO_2_: partial pressure pf Oxygen, PaCO_2_: partial pressure of carbon dioxide.

**Table 3:** shows comparison of follow-up recommendations between 1-4 weeks and 12-16 weeks in GOLD 2023 report. Only difference has been highlighted in a different color. Adapted from GOLD 2023.

Post Bronchodilator FEV1/FVC < 0.7 in COPD Patients	
GOLD 1	FEV1 ≥ 80%
GOLD 2	FEV1 ≥ 50% and < 80%
GOLD 3	FEV1 ≥ 30% and < 50%
GOLD 4	FEV1 <30%

**Table 4:** A simple adaptation of grades of airflow obstruction in COPD (based on post-bronchodilator FEV1).

1-4 Weeks Follow-up	12-16 Weeks Follow-up
-Evaluation of patient’s ability to cope in current environment -Review patient’s understanding about his/her COPD treatment regimen -Reassess inhaler technique -Review need for long term oxygen therapy -Reassess the need for pulmonary rehabilitation -Document CAT or mMRC score -Analyze comorbidities	-Evaluation of patient’s ability to cope in current environment -Review patient’s understanding about his/her COPD treatment regimen -Reassess inhaler technique -Review need for long term oxygen therapy -Reassess the need for pulmonary rehabilitation -document CAT or mMRC score -Analyze comorbidities -Obtain Spirometry measurement (FEV1).

## LIST OF ABBREVIATIONS

GOLD: Chronic Obstructive Lung Disease
COPD: Chronic Obstructive Lung Disease
FVC: Forced Vital Capacity
FEV1: Forced Expiratory Volume in one second
CAT: COPD Assessment Tool
ICS: Inhaled Corticosteroid
